# Supplementary material for: Resting brain dynamics at different timescales capture distinct aspects of human behavior
Source: Nat Commun. 2019 May 24;10:2317. doi: 10.1038/s41467-019-10317-7 (PMC6534566; doi:10.1038/s41467-019-10317-7)
Supplement: Supplementary file 1 — Supplementary Information [file 41467_2019_10317_MOESM1_ESM.pdf]

Supplementary Information

Supplementary Methods

Estimation of multidimensional variance component model

The multidimensional variance component model of Ge and colleagues [1] writes as follows if a single kernel is used (i.e., if either a static similarity matrix or a dynamic similarity matrix is considered):

$$Y = C + E, \tag{1}$$

where Y, C and E are 419×58 matrices. Y contains the 58 processed behavioral measures for all 419 subjects. $Vec(C) \sim \mathcal{N}(0, \Sigma_c \otimes F)$ and $Vec(E) \sim \mathcal{N}(0, \Sigma_e \otimes I)$, where $Vec(.)$ is the matrix vectorization operator, $\otimes$ is the Kronecker product of matrices, and I is the identity matrix. F is a similarity matrix such that $F(i, j)$ encodes the (static or dynamic) FC similarity between subjects i and j, and is defined as the correlation between the static FC (or dynamic FC) matrices of the two subjects. Σ_c and Σ_e are unknown 58×58 matrices to be estimated from F and Y. Estimates of Σ_c and Σ_e are obtained using a moment-matching method [1]:

$$\hat{\Sigma}_c = \frac{1}{\nu_F} Y^T (F - \tau I) Y \quad \text{and} \quad \hat{\Sigma}_e = \frac{1}{\nu_F} Y^T (\kappa I - \tau F) Y, \tag{2}$$

where $\tau = Tr(F)/N$, $\kappa = Tr(F^2)/N$, and $\nu_F = N(\kappa - \tau^2)$. The variance explained by (static or dynamic) FC markers, denoted M, then writes:

$$M = \frac{Tr(\Sigma_c)}{Tr(\Sigma_c) + Tr(\Sigma_e)}. \tag{3}$$

Variance explained for a single behavioral measure is given by $M_i = \Sigma_c(i,i)/(\Sigma_c(i,i) + \Sigma_e(i,i))$. If more than one kernel is used in the analysis (e.g., if one wants to explore the variance explained when static and dynamic FC are combined), Supplementary Eq. (1) generalizes as follows:

$$Y = \sum_l C_l + E, \tag{4}$$

where $Vec(C_l) \sim \mathcal{N}(0, \Sigma_{c_l} \otimes F_l)$ and $Vec(E) \sim \mathcal{N}(0, \Sigma_e \otimes I)$. The variance explained by all components is defined as:

$$M = \frac{\sum_l Tr(\Sigma_{c_l})}{\sum_l Tr(\Sigma_{c_l}) + Tr(\Sigma_e)}. \tag{5}$$

The variance explained by a particular component C_{l_0} is defined as:

$$M_{l_0} = \frac{Tr(\Sigma_{c_{l_0}})}{\sum_l Tr(\Sigma_{c_l}) + Tr(\Sigma_e)}, \tag{6}$$

and the variance explained for a single behavioral measure i is computed as:

$$M_i = \frac{\sum_l \Sigma_{C_l}(i,i)}{\sum_l \Sigma_{C_l}(i,i) + \Sigma_e(i,i)}. \tag{7}$$

Estimates of Σ_{C_l} and Σ_e are now computed as follows. Denoting the (r,s)-element of Σ_{C_l} as $\sigma_{C_l,rs}$ and the (r,s)-element of Σ_e as $\sigma_{e,rs}$, we have:

$$cov(y_r, y_s) = \sum_l \sigma_{C_l,rs} F_l + \sigma_{e,rs} I. \tag{8}$$

We then regress $Vec(y_r y_s^T)$ onto $Vec(F_l)$ and $Vec(I)$ which leads to the following linear system:

$$\begin{bmatrix} Tr(F_1F_1) & \dots & Tr(F_1F_L) & Tr(F_1) \\ \vdots & \ddots & \vdots & \vdots \\ Tr(F_LF_1) & \dots & Tr(F_LF_L) & Tr(F_L) \\ Tr(F_1) & \dots & Tr(F_L) & Tr(I) \end{bmatrix} \sigma_{rs} = \begin{bmatrix} y_r^T F_1 y_s \\ \vdots \\ y_r^T F_L y_s \\ y_r^T y_s \end{bmatrix}, \tag{9}$$

where $\sigma_{rs} = (\sigma_{C_1,rs}, \dots, \sigma_{C_L,rs}, \sigma_{e,rs})^T$. Solving the linear system gives the (r,s)-element in each of the variance component matrices, and Σ_c and Σ_e are estimated by repeating this for all r and s. Note that when only one kernel is used, a closed-form estimator can be derived, which is Supplementary Eq. (2).

The mean and standard deviation (SD) of the behavioral variance explained by -static or dynamic- FC patterns are then computed using the Jackknife method (Equations (4) and (5); [2]).

Importantly, the estimated SD of the 'delete-1' estimates is not the SD of the behavioral variance represented by error bars in Figures 1-4, as can be seen from Equation (5). More precisely, the SD of the delete-1 estimates is (much) smaller than the SD of the explained variance. This is explained by the fact that the delete-1 estimates are computed from subsets of size $(N-1)$ sharing all but one subject, and hence the delete-1 estimates are close to each other. This redundancy is taken into account in Equation (5) to compute the SD of the explained variance. For these reasons, the delete-1 estimates can not be overlaid as dots onto the bar charts shown in Figures 1-4. First, the reduced range would make the dots hard to visualise. Second -and most importantly-, as detailed here above the error bars of our bar charts represent the SD of the estimated explained variance and do not represent the SD of the delete-1 estimates. It would therefore be misleading to plot both delete-1 estimates and the SD estimate of the explained variance (error bars in our Figures) because they are only indirectly related through Equation (5).

Identifying patterns of interactions contributing to the overall explained variance

The contribution of pairwise interactions to the overall explained variance is obtained from the following model:

$$y = Wu + e, \tag{10}$$

where y is a vector encoding one behavioral measure for the N subjects, u is a random-effects vector of length P, the number of entries in the FC matrices, W is an $N \times P$ matrix with centered and unit-variance lines, and e is the normally distributed residual with variance σ_e. Assuming each element of u is independent and follows a normal distribution with variance σ_c/P, then the model can be turned into the variance component model of Equation (2) we used: $Cov(y) = \sigma_c \cdot F + \sigma_e \cdot I$, where $F = W \cdot W^T/P$ is the similarity matrix of the connectome between pairs of individuals. Then, using the best linear unbiased predictor of u following Yang et al. (2011) [3], the entries of u^2 provide a scaled estimate of the variance explained by each pair of ROIs. To evaluate the contributions of ROI pairs over all behavioral measures of task performance, u^2 was computed for each task-related behavioral measure and weighted by the loadings of the first principal component of these behavioral measures. This weighted u^2 was used to produce Figure 5, as further detailed

in the Supplementary Results (Supplementary Figure 4).

Accounting for covariates

Age, gender, race, education and motion (mean FD) were regressed from the 58 phenotypic measures which were then quantile normalized. To do so, each behavioral measure distribution was sorted and mapped to a linear spacing of the $]0, 1[$ interval. Each behavioral measure was then replaced by the inverse normal cdf of its mapped value, leading to a rank-preserving Gaussian redistribution of the behavioral measures [4]. This normalization was motivated by the fact that Gaussianity is an assumption of the multidimensional variance component model. Quantile normalization, however, should not be applied on distributions that are too 'exotic' (too skewed, too few values, etc.). To verify this, we inspected the distribution of all the behavioral measures. Among the 58 measures, none are binary, 18 are ordinal (take only integer values), 40 are continuous, and all are reasonably close to the Gaussian distribution (based on visual inspection of the histograms, and computation of skewness and kurtosis for each measure). We finally note that results were not significantly affected if no quantile normalization was performed.

An alternative way of including covariates would have been to explicitly account for them in the variance component model:

$$Y = XB + C + E, \tag{11}$$

where X is an $N \times Q$ matrix of Q covariates and B a matrix of fixed effects. Again, using this alternative approach did not significantly affect our results.

Supplementary Results

Variance explained for additional behavioral measures

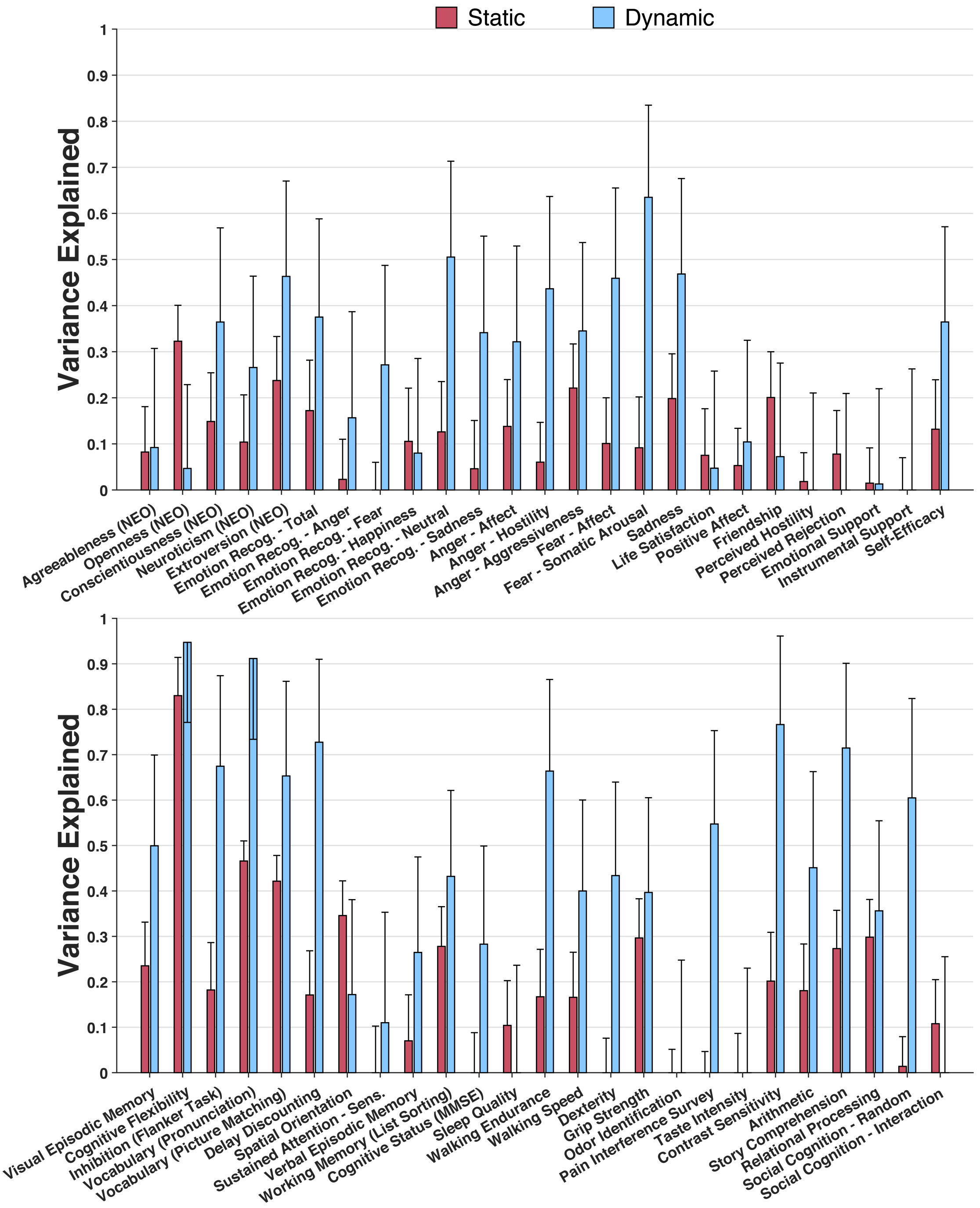

Supplementary Figure 1: Variance explained for 50 among the 58 HCP behavioral measures. Static FC utilizes Pearson's correlation, while dynamic FC utilizes the coefficient matrix of a first-order autoregressive model. Error bars indicate SD of the estimates.

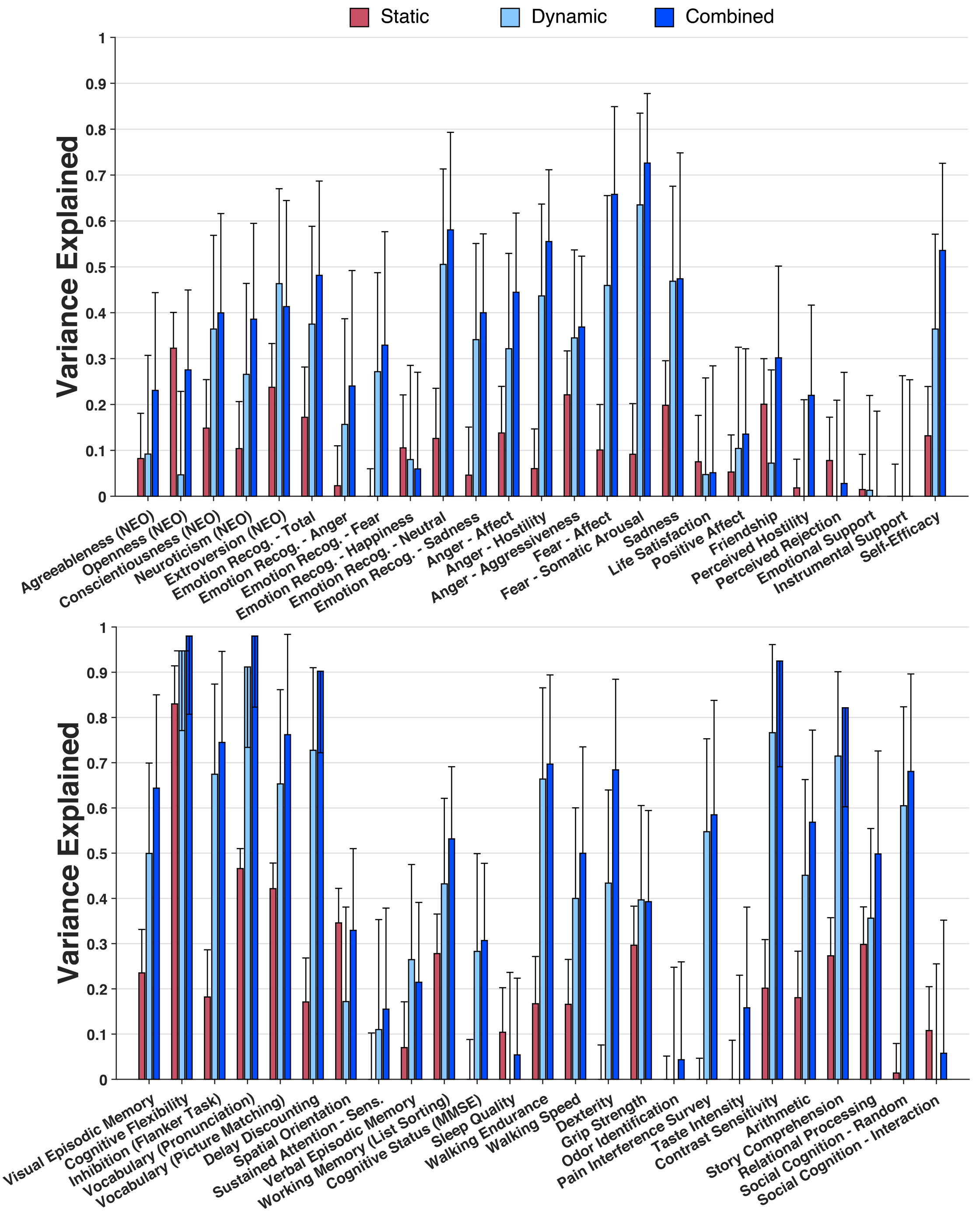

Supplementary Figure 2: Variance explained for 50 among the 58 HCP behavioral measures, including combined model. Static FC utilizes Pearson's correlation, while dynamic FC utilizes the coefficient matrix of a first-order autoregressive model. Error bars indicate SD of the estimates. The variance explained when combining static and dynamic FC is also shown (dark blue).

Exploring subcategories of task-based measures

To test whether the advantage of dynamic FC in explaining task-based behavioral measures was shared across different types of task-based measures, we computed the behavioral variance explained by dynamic and static FC in subcategories of task-based measures. These categories were determined based on the the expected cognitive domains recruited by the tasks [5]. We merged 'Social' and 'Memory' measures in order to avoid under-represented categories and also because the corresponding experiments are expected to recruit overlapping networks such as the default mode network, leading to the following partitioning:

- Executive Function: Cognitive Flexibility; Fluid Intelligence; Working Memory (N-back); Working Memory (List Sorting); Relational Processing; Arithmetic; Inhibition (Flanker Task). (8)
- Emotion: Emotion Recog. – Total; Emotion Recog. – Anger; Emotion Recog. – Fear; Emotion Recog. – Happiness; Emotion Recog. – Neutral; Emotion Recog. – Sadness; Emotion face matching. (7)
- Memory/Social: Visual Episodic Memory;Verbal Episodic Memory; Social Cognition – Random; Social Cognition – Interaction. (4)
- Visuo-spatial Attention: Sustained Attention – Sens.; Sustained Attention – Spec.; Processing Speed; Spatial Orientation. (4)
- Language: Vocabulary (Pronunciation); Vocabulary (Picture Matching); Story Comprehension. (3)
- Unclassified: Dexterity. (1)

Supplementary Figure 3 shows that the main finding is preserved, even if in some subcategories the difference between static and dynamic explained variance does not reach statistical significance ($p > 0.05$, two-tailed t-test) which might be due to the limited number of measures composing these categories. These results strengthen our findings by showing that the better capacity of dynamic FC to explain task-based behavioral variance is reproduced in several subtypes of task-based measures.

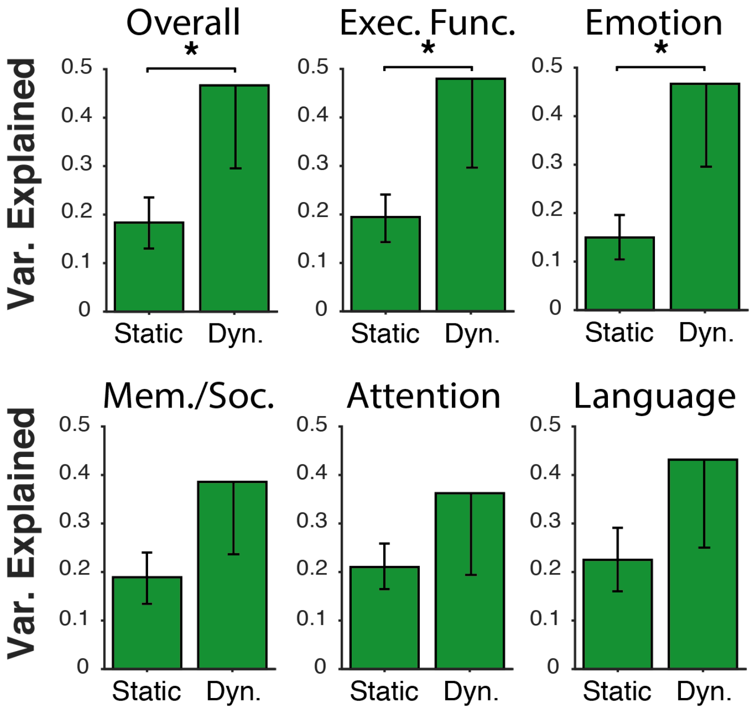

Supplementary Figure 3: Behavioral variance explained by static and dynamic FC in all ('Overall') and subcategories of task-based measures: Executive Function, Emotion, Memory/Social, Visuo-spatial Attention, and Language. Error bars indicate SD of the estimates.

Dynamic FC interactions most contributing to task-behavioral explained variance

Supplementary Figure 4 shows the pairs of ROIs of the dynamic FC pattern most contributing to the task-behavioral explained variance, obtained from Supplementary Equation (10). This result is used to generate Figure 5 by averaging contributions in the network-interactions using the 17-network parcellation [5]. To explore whether the explained variance was concentrated in some network interactions, we compared these values to the ones obtained after randomly shuffling lines and columns in Supplementary Figure 4. The interactions shown in Figure 5 are the ones surviving an FDR correction at the level $q = 0.05$.

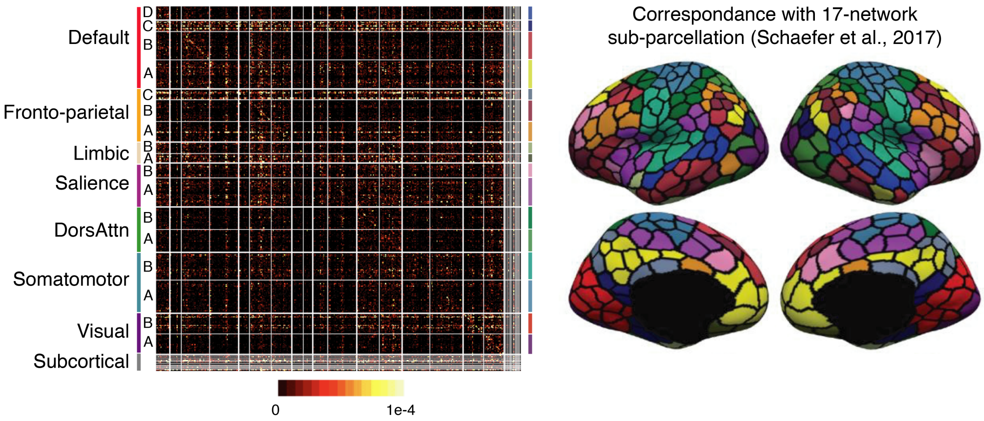

Supplementary Figure 4: (Left) Contribution of each pairwise ROI interaction to task-behavioral explained variance, following Supplementary Equation (10). The color code corresponds to the 7-network parcellation used in Figures 3 and 5. (Right) Correspondance with 17-network sub-parcellation [5].

Results on replication dataset

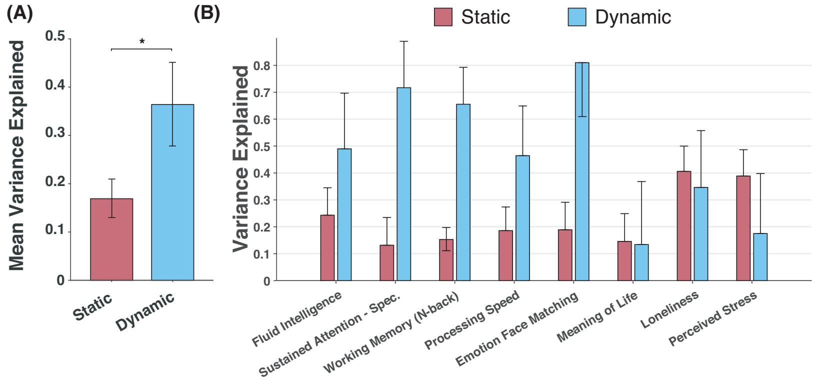

Supplementary Figure 5: Replication of results from Figure 1 on the replication dataset containing 328 subjects, with same analyses procedures and color code. Error bars indicate SD of the estimates.

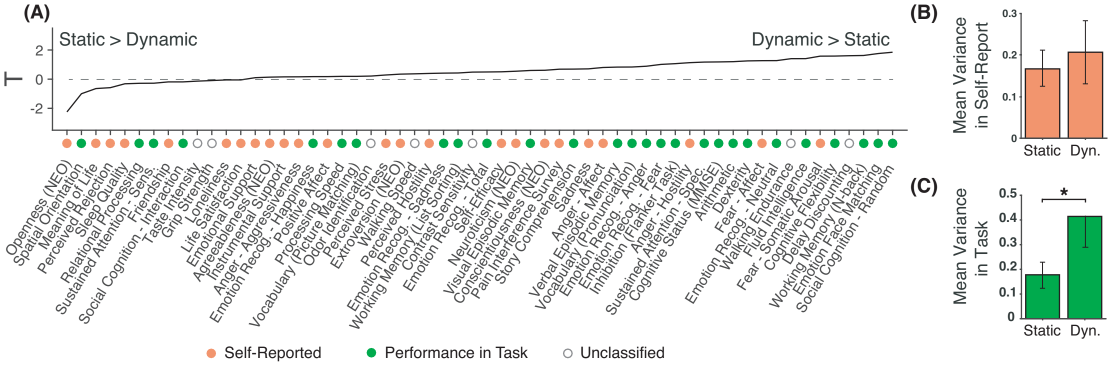

Supplementary Figure 6: Replication of results from Figure 2 on the replication dataset containing 328 subjects, with same analyses procedures and color code. Error bars indicate SD of the estimates.

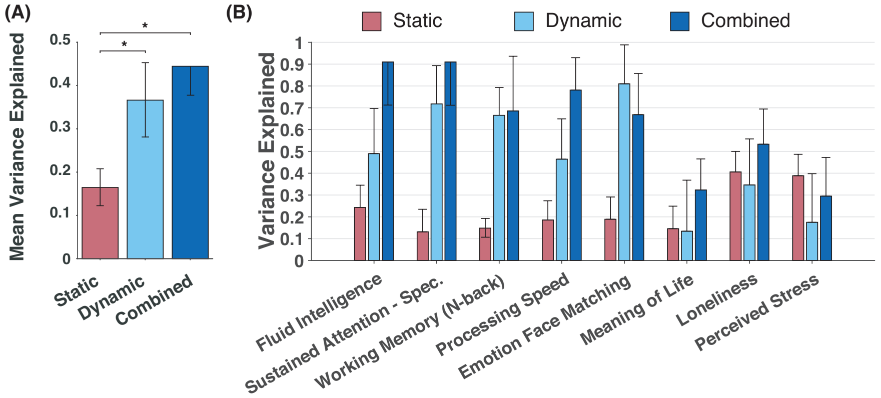

Supplementary Figure 7: Replication of results from Figure 4 on the replication dataset containing 328 subjects, with same analyses procedures and color code. Error bars indicate SD of the estimates.

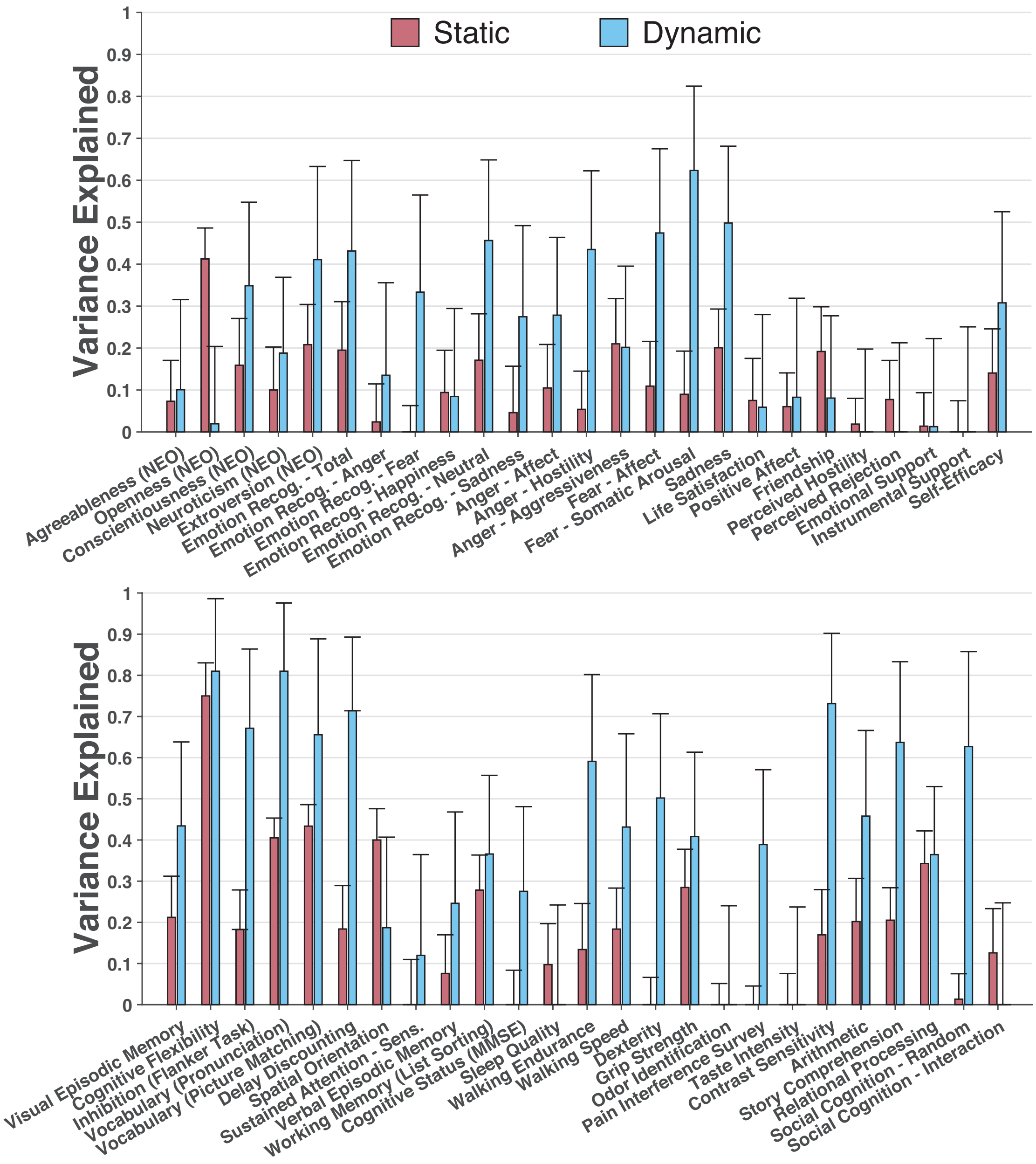

Supplementary Figure 8: Replication of results from Supplementary Figure 1 on the replication dataset containing 328 subjects, with same analyses procedures and color code. Error bars indicate SD of the estimates.

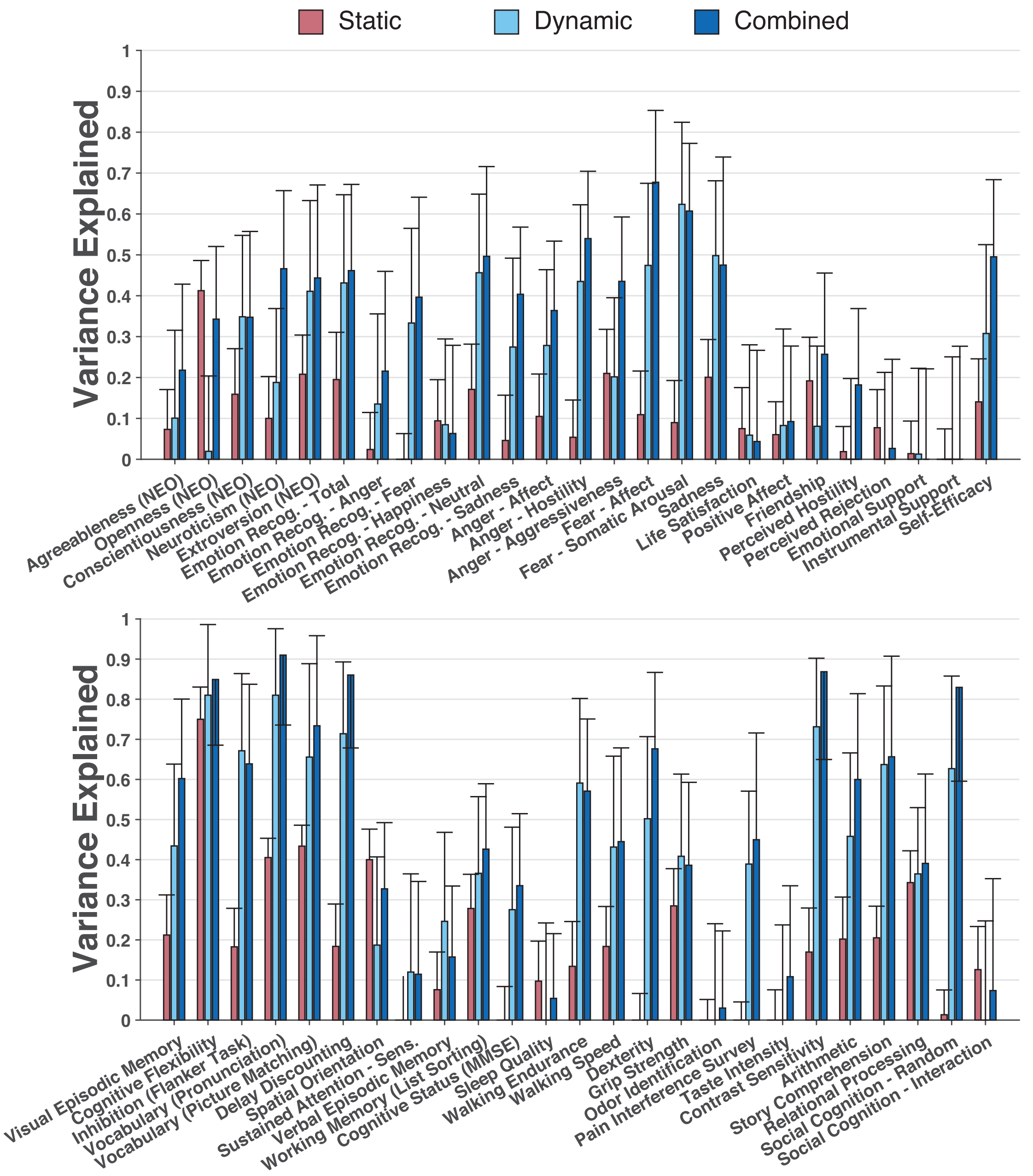

Supplementary Figure 9: Replication of results from Supplementary Figure 2 on the replication dataset containing 328 subjects, with same analyses procedures and color code. Error bars indicate SD of the estimates.

Supplementary Table 1 reports the p-values of the statistical tests (two-tailed t-tests) performed in Figures 1-4 (original dataset) and the corresponding tests in the replication dataset (Supplementary Figures 5-9). The p-values marked with an asterix are the ones surviving an FDR correction at the level $q < 0.05$, when correcting for the 16 tests reported in Supplementary Table 1.

	**Test**	**p-value (orig.)**	**p-value (repl.)**
1	Mean static vs. dynamic (Fig. 1)	$8.31 \times 10^{-4*}$	$2.30 \times 10^{-3*}$
2	Static vs. dynamic in Self-Report (Fig. 2B)	2.52×10^{-1}	3.14×10^{-1}
3	Static vs. dynamic in Task (Fig. 2C)	$1.75 \times 10^{-3*}$	$2.51 \times 10^{-3*}$
4	Interaction effect (Figs. 2B&C)	$3.62 \times 10^{-3*}$	$4.30 \times 10^{-3*}$
5	Static vs. dynamic within networks (Fig. 3)	4.51×10^{-1}	3.14×10^{-1}
6	Static vs. dynamic between networks (Fig. 3)	$8.31 \times 10^{-3*}$	$2.16 \times 10^{-2*}$
7	Mean static vs. combined (Fig. 4)	$4.73 \times 10^{-4*}$	$1.15 \times 10^{-4*}$
8	Mean dynamic vs. combined (Fig. 4)	2.89×10^{-1}	3.91×10^{-1}

Table 1: List of statistical tests performed and corresponding p-values in original (orig.; 419 subjects) and replication (repl.; 328 subjects) datasets. The asterisk denotes p-values that survived FDR correction at $q < 0.05$.

Additional control analyses

We performed four control analyses to evaluate the impact of different processing steps included in our baseline analysis:

1. Including the variance of the mean grayordinate signal as a covariate in the variance component model (Supplementary Figure 10B).
2. Computing the static and dynamic FC matrices from fMRI time series on which no mean grayordinate signal was performed (Supplementary Figure 10C).
3. Including head motion metrics (mean FWD, max FWD and number of volumes scrubbed) as covariates of the variance component model (Supplementary Figure 10D).
4. Computing the static and dynamic FC matrices from full (i.e., uncensored) fMRI time series (Supplementary Figure 10E).

In each variant, the main results are reproduced.

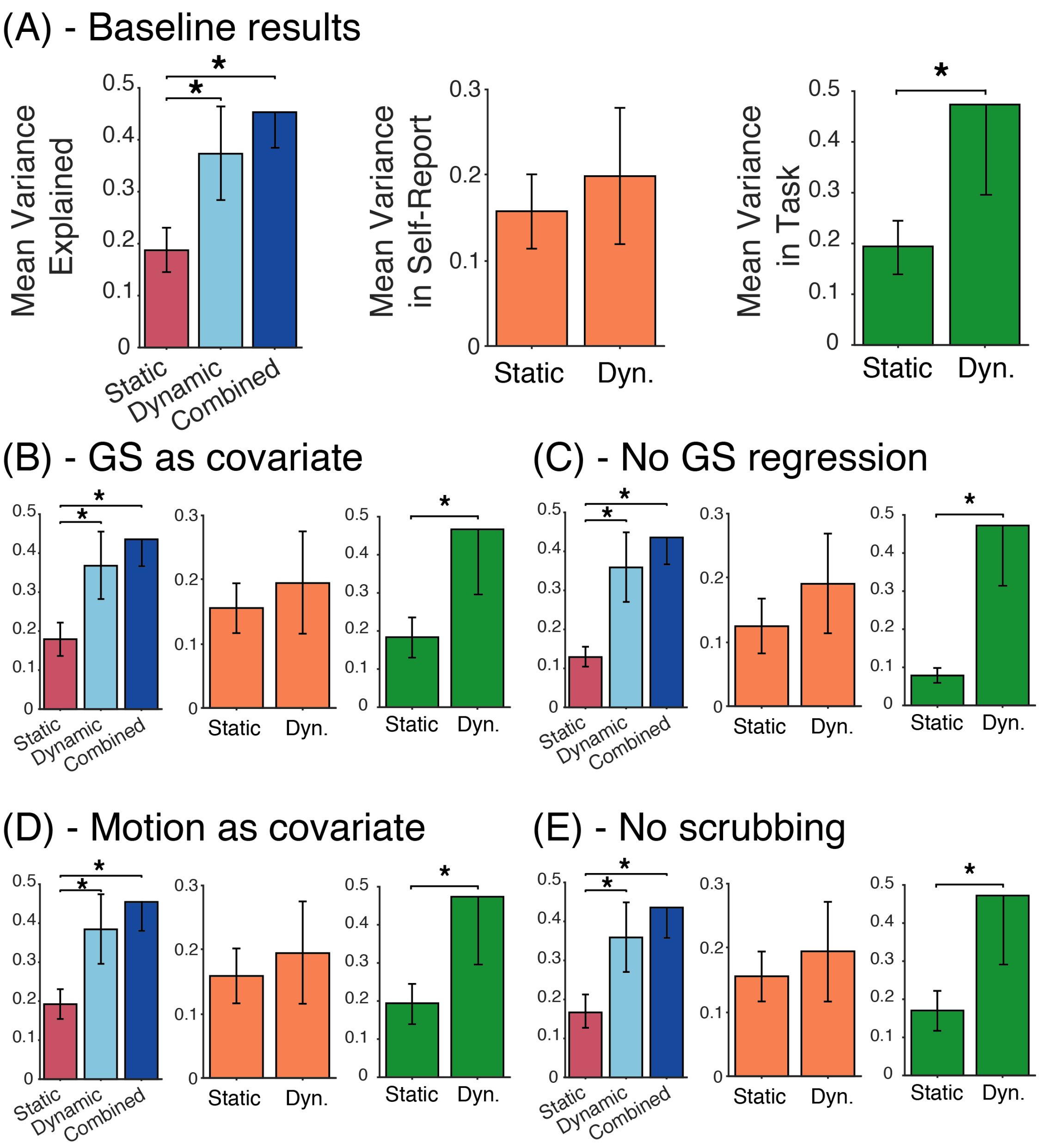

Supplementary Figure 10: Primary findings are reproduced in different variants of the preprocessing setup. (A) Main original results. (B) Main results when including the variance of mean grayordinate signal as a covariate. (C) Main results when the static and dynamic FC matrices are computed from fMRI time series on which no mean grayordinate signal was performed. (D) Main results when including head motion metrics (mean FWD, max FWD and number of volumes scrubbed) as covariates of the variance component model. (E) Main results when the static and dynamic FC matrices are computed from full (i.e., uncensored) fMRI time series.

We also tested the effect of the number of dimensions considered in the variance component model. We tested this effect both on average over different measures (Supplementary Figure 11A), and for individual measures (Supplementary Figure 11B-C). In the first case we randomly selected

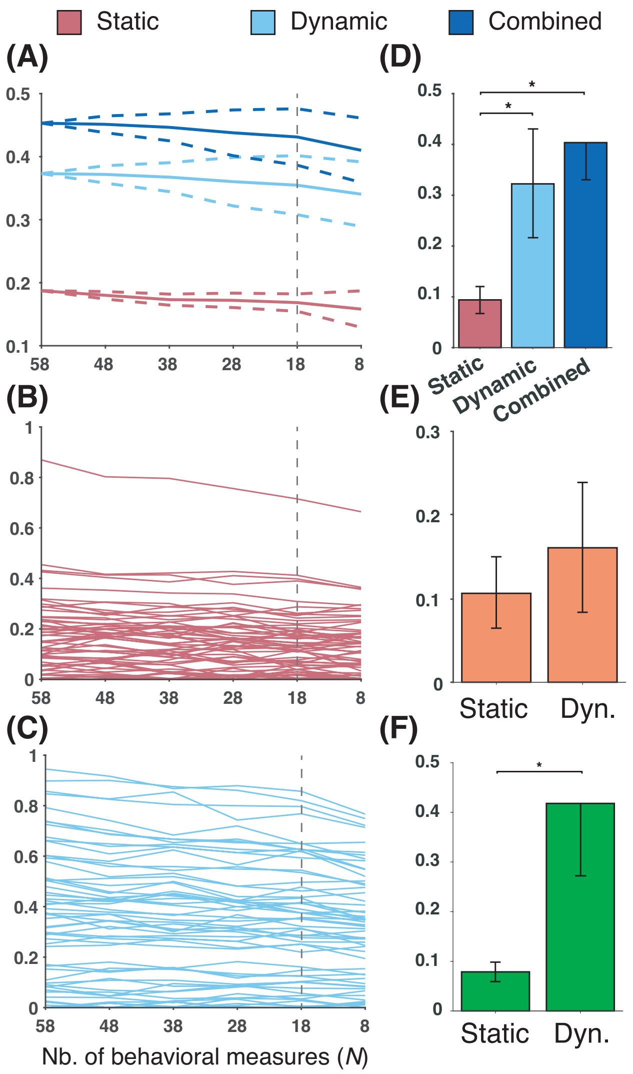

Supplementary Figure 11: Impact of the number of dimensions considered in the variance component model. (A) Mean (plain) and standard deviation (dashed) of the average explained variance over N randomly chosen dimensions using 100 samples. (B-C) Mean explained variance for all individual dimensions computed using $N-1$ other randomly chosen dimensions in the static (B) and dynamic (C) cases. (D-F) Reproduction of our main results using $N = 18$ (dashed gray line). The mean and standard deviations are computed from the 100 point estimates and not through the Jackknife approach. Error bars indicate SD of the estimates.

N behavioral measures and computed the mean (and standard deviation) point estimate over 100 random selections of these N behavioral measures. In the second case, for each behavioral measure we ran a model including this behavioral measure plus $N - 1$ other randomly chosen behavioral measures. This operation was repeated 100 times for each behavioral measure and the mean (and standard deviation) point estimates are shown in Supplementary Figure 11B-C. We finally verified that our main findings are reproduced for the special case of $N = 18$ (Suppl. Figures 11D-F).

Static and dynamic contribution in the combined model

We present the relative contributions of static and dynamic FC variance in the combined model used in Figure 4. It can be seen that as in the case of individual models, dynamic FC captures more behavioral variance than static FC within the combined model: out of the average 45% explained by the combined model, 12% are attributed to static FC and 33% to dynamic FC (Supplementary Figure 12A). Note that as the combined explained variance (45%) is smaller than the sum of individual static (18%) and dynamic (37%) explained variances, there is shared variance between the static and dynamic contributions. Further work is required to determine how this shared variance is distributed among various contributions from a theoretical point of view and hence this result should be considered with caution.

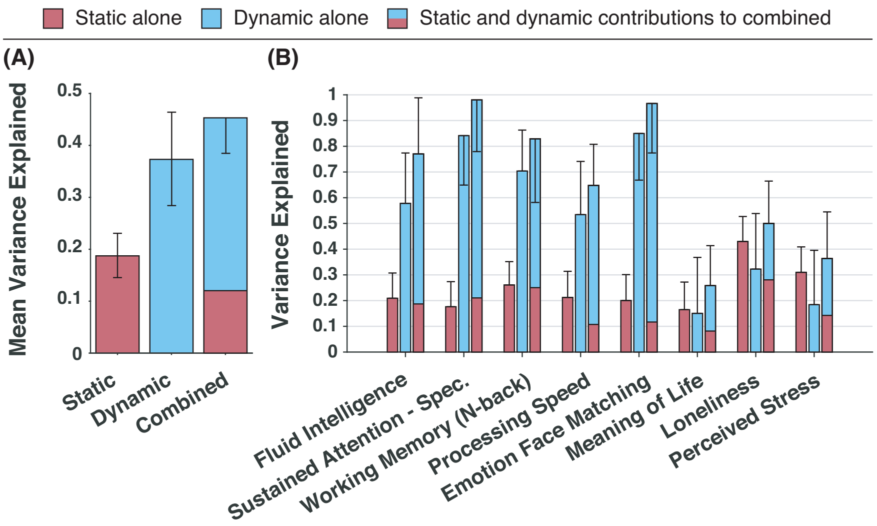

Supplementary Figure 12: Comparison between explained variances in individual models (first two bars of each group) and distribution of variance in combined model (third bar of each group). (A) Average over 58 behavioral measures. (B) Results for 8 individual behavioral measures. Error bars indicate SD of the estimates.

	HCP Field	Friendly Name	Class		HCP Field	Friendly Name	Class
1.	PicSeq Unadj	Visual Episodic Memory	TA	30.	WM Task Acc	Working Memory (N-back)	TA
2.	CardSort Unadj	Cognitive Flexibility	TA	31.	NEOFAC A	Agreeableness (NEO)	SR
3.	Flanker Unadj	Inhibition (Flanker Task)	TA	32.	NEOFAC O	Openness (NEO)	SR
4.	PMAT24 A CR	Fluid Intelligence	TA	33.	NEOFAC C	Conscientiousness (NEO)	SR
5.	ReadEng Unadj	Vocabulary (Pronunciation)	TA	34.	NEOFAC N	Neuroticism (NEO)	SR
6.	PicVocab Unadj	Vocabulary (Picture Matching)	TA	35.	NEOFAC E	Extroversion (NEO)	SR
7.	ProcSpeed Unadj	Processing Speed	TA	36.	ER40 CR	Emotion Recog. – Total	TA
8.	DDisc AUC 40K	Delay Discounting	UC	37.	ER40ANG	Emotion Recog. – Anger	TA
9.	VSPLOT TC	Spatial Orientation	TA	38.	ER40FEAR	Emotion Recog. – Fear	TA
10.	SCPT SEN	Sustained Attention – Sens.	TA	39.	ER40HAP	Emotion Recog. – Happiness	TA
11.	SCPT SPEC	Sustained Attention – Spec.	TA	40.	ER40NOE	Emotion Recog. – Neutral	TA
12.	IWRD TOT	Verbal Episodic Memory	TA	41.	ER40SAD	Emotion Recog. – Sadness	TA
13.	ListSort Unadj	Working Memory (List Sorting)	TA	42.	AngAffect Unadj	Anger - Affect	SR
14.	MMSE Score	Cognitive Status (MMSE)	TA	43.	AngHostil Unadj	Anger - Hostility	SR
15.	PSQI Score	Sleep Quality	SR	44.	AngAggr Unadj	Anger - Aggressiveness	SR
16.	Endurance Unadj	Walking Endurance	UC	45.	FearAffect Unadj	Fear - Affect	SR
17.	GaitSpeed Comp	Walking Speed	UC	46.	FearSomat Unadj	Fear - Somatic Arousal	SR
18.	Dexterity Unadj	Dexterity	TA	47.	Sadness Unadj	Sadness	SR
19.	Strength Unadj	Grip Strength	UC	48.	LifeSatisf Unadj	Life Satisfaction	SR
20.	Odor Unadj	Odor Identification	UC	49.	MeanPurp Unadj	Meaning of Life	SR
21.	PainInterf Tscore	Pain Interference Survey	SR	50.	PosAffect Unadj	Positive Affect	SR
22.	Taste Unadj	Taste Intensity	UC	51.	Friendship Unadj	Friendship	SR
23.	Mars Final	Contrast Sensitivity	UC	52.	Loneliness Unadj	Loneliness	SR
24.	Emotion Task Face Acc	Emotion Face Matching	TA	53.	PercHostil Unadj	Perceived Hostility	SR
25.	Lang. Task Math Av Diff	Arithmetic	TA	54.	PercReject Unadj	Perceived Rejection	SR
26.	Lang. Task Story Av Diff	Story Comprehension	TA	55.	EmotSupp Unadj	Emotional Support	SR
27.	Relational Task Acc	Relational Processing	TA	56.	InstruSupp Unadj	Instrumental Support	SR
28.	Social Task Perc Rand	Social Cognition – Random	TA	57.	PercStress Unadj	Perceived Stress	SR
29.	Social Task Perc TOM	Social Cognition – Interaction	TA	58.	SelfEff Unadj	Self-Efficacy	SR

Table 2: List of the 58 behavioral measures from the Human Connectome Project used in the present work. These measures were selected so as to span cognitive, emotion and social behavioral aspects and were classified as task performance measures (TA), self-reported measures (SR), or left unclassified (UC).

Supplementary References

[1] Ge, T. *et al.* Multidimensional heritability analysis of neuroanatomical shape. *Nat Commun* **7**, 13291 (2016).

[2] Efron, B. The jackknife, the bootstrap and other resampling plans. *SIAM* **38** (1982).

[3] Yang, J., Lee, S. H., Goddard, M. E. & Visscher, P. M. Gcta: a tool for genome-wide complex trait analysis. *Am J Hum Genet* **88**, 76–82 (2011).

[4] Elliott, L. T. *et al.* Genome-wide association studies of brain imaging phenotypes in uk biobank. *Nature* **562**, 210–216 (2018).

[5] Barch, D. M. *et al.* Function in the human connectome: task-fmri and individual differences in behavior. *Neuroimage* **80**, 169–89 (2013).

[6] Schaefer, A. *et al.* Local-global parcellation of the human cerebral cortex from intrinsic functional connectivity mri. *Cerebral Cortex* **28**, 3095–114 (2018).
