# Supplementary material for: Resting brain dynamics at different timescales capture distinct aspects of human behavior
Source: Nat Commun. 2019 May 24;10:2317. doi: 10.1038/s41467-019-10317-7 (PMC6534566; doi:10.1038/s41467-019-10317-7)
Supplement: Supplementary file 3 — Reporting Summary [file 41467_2019_10317_MOESM3_ESM.pdf]

nature research

Corresponding author(s): R. Liégeois & B.T.T. Yeo

Last updated by author(s): Apr 26, 2019

Reporting Summary

Nature Research wishes to improve the reproducibility of the work that we publish. This form provides structure for consistency and transparency in reporting. For further information on Nature Research policies, see Authors & Referees and the Editorial Policy Checklist.

Statistics

For all statistical analyses, confirm that the following items are present in the figure legend, table legend, main text, or Methods section.

n/a	Confirmed	
☐	☒	The exact sample size (*n*) for each experimental group/condition, given as a discrete number and unit of measurement
☐	☒	A statement on whether measurements were taken from distinct samples or whether the same sample was measured repeatedly
☐	☒	The statistical test(s) used AND whether they are one- or two-sided<br>*Only common tests should be described solely by name; describe more complex techniques in the Methods section.*
☐	☒	A description of all covariates tested
☐	☒	A description of any assumptions or corrections, such as tests of normality and adjustment for multiple comparisons
☐	☒	A full description of the statistical parameters including central tendency (e.g. means) or other basic estimates (e.g. regression coefficient) AND variation (e.g. standard deviation) or associated estimates of uncertainty (e.g. confidence intervals)
☐	☒	For null hypothesis testing, the test statistic (e.g. *F*, *t*, *r*) with confidence intervals, effect sizes, degrees of freedom and *P* value noted<br>*Give P values as exact values whenever suitable.*
☒	☐	For Bayesian analysis, information on the choice of priors and Markov chain Monte Carlo settings
☒	☐	For hierarchical and complex designs, identification of the appropriate level for tests and full reporting of outcomes
☐	☒	Estimates of effect sizes (e.g. Cohen's *d*, Pearson's *r*), indicating how they were calculated

Our web collection on statistics for biologists contains articles on many of the points above.

Software and code

Policy information about availability of computer code

Data collection	We used data from the publicly available Human Connectome Dataset.
Data analysis	Data was analyzed using Matlab code (versions 2014a and 2016a)

For manuscripts utilizing custom algorithms or software that are central to the research but not yet described in published literature, software must be made available to editors/reviewers. We strongly encourage code deposition in a community repository (e.g. GitHub). See the Nature Research guidelines for submitting code & software for further information.

Data

Policy information about availability of data

All manuscripts must include a data availability statement. This statement should provide the following information, where applicable:

- Accession codes, unique identifiers, or web links for publicly available datasets
- A list of figures that have associated raw data
- A description of any restrictions on data availability

The HCP data is publicly available at http://www.humanconnectomeproject.org/data/; informed consent was obtained from all HCP participants (Van Essen et al., 2012).

Field-specific reporting

Please select the one below that is the best fit for your research. If you are not sure, read the appropriate sections before making your selection.

☒ Life sciences ☐ Behavioural & social sciences ☐ Ecological, evolutionary & environmental sciences

Life sciences study design

All studies must disclose on these points even when the disclosure is negative.

Sample size	Original sample size is N=419 and was determined by selecting the largest number of unrelated HCP subjects
Data exclusions	HCP subjects with no fMRI runs meeting the preprocessing requirements (in particular regarding the level of motion, see hereunder) or missing behavioral measures were excluded.
Replication	The replication dataset contains 328 subjects corresponding to the second subject of each HCP family containing more than one person. We not that it is therefore not completely independent from the original dataset.
Randomization	No groups are defined within subjects. Age, gender, race, education and motion (mean FD) were regressed from the behavioral measures.
Blinding	Blinding is not relevant to this study as no data collection was involved

Reporting for specific materials, systems and methods

We require information from authors about some types of materials, experimental systems and methods used in many studies. Here, indicate whether each material, system or method listed is relevant to your study. If you are not sure if a list item applies to your research, read the appropriate section before selecting a response.

Materials & experimental systems

n/a	Involved in the study	
☒	☐	Antibodies
☒	☐	Eukaryotic cell lines
☒	☐	Palaeontology
☒	☐	Animals and other organisms
☐	☒	Human research participants
☒	☐	Clinical data

Methods

n/a	Involved in the study	
☒	☐	ChIP-seq
☒	☐	Flow cytometry
☐	☒	MRI-based neuroimaging

Human research participants

Policy information about studies involving human research participants

Population characteristics	Healthy adults (ages 22 - 35) drawn from a population of siblings. Out of the 747 subjects used in the study (original + replication), 420 are females
Recruitment	N.A., see Van Essen et al., 2012.
Ethics oversight	Consortium of institutions in the US and Europe, led by Washington University and the University of Minnesota (the 'WU-Minn HCP Consortium')

Note that full information on the approval of the study protocol must also be provided in the manuscript.

Magnetic resonance imaging

Experimental design

Design type	Only resting-state fMRI data was used
Design specifications	The duration of each fMRI run is 14.4 min (1200 volumes, TR=0.72 sec)
Behavioral performance measures	No behavioral measures were acquired during the fMRI scan recordings

Acquisition

Imaging type(s)	Functional & Structural
Field strength	3T
Sequence & imaging parameters	Multi-band sequence; functional images have a 2-mm isotropic spatial resolution whereas structural images are 0.7-mm isotropic.
Area of acquisition	Whole brain scans
Diffusion MRI	☐ Used ☒ Not used

Preprocessing

Preprocessing software	We used the HCP preprocessed data; preprocessing softwares included FSL 5.0.6, FreeSurfer 5.3.0-HCP, and Connectome Workbench v1.1.1.
Normalization	Multimodal surface matching (MSM; Robinson et al. 2014, DOI:10.1016/j.neuroimage.2014.05.069) and nonlinear transformation were used
Normalization template	We used the fs_LR_32k surface mesh for normalization
Noise and artifact removal	FMRIB's ICA-based X-noiseifier (FIX), global signal regression
Volume censoring	Customized MATLAB scripts were utilized to select censored frames. First, volumes with FD > 0.2mm or DVARS > 75 were marked as outliers. Second, one frame before and two frames after these volumes were also flagged as outliers. Finally, remaining segments of data that lasted fewer than five contiguous volumes were censored. BOLD runs with more than half of the frames flagged as censored were removed.

Statistical modeling & inference

Model type and settings	We use the multidimensional variance component model of Ge et al. (2016)
Effect(s) tested	We test whether static or dynamic (resting-state) FC patterns are associated with various phenotypic measures

Specify type of analysis: ☐ Whole brain ☒ ROI-based ☐ Both

Anatomical location(s)	We used the atlas defined in Schaefer et al. (2018)
Statistic type for inference (See Eklund et al. 2016)	The whole-brain FC patterns are used in the association analysis; Associations with phenotypic measures were computed using the delete-1 Jackknife approach (Efron, 1982)
Correction	We accounted for multiple comparisons using FDR corrections

Models & analysis

n/a	Involved in the study
☐	☒ Functional and/or effective connectivity
☒	☐ Graph analysis
☐	☒ Multivariate modeling or predictive analysis

Functional and/or effective connectivity	Functional connectivity is measured using Pearson correlation of the fMRI time series (static measures) and the model-parameter of a 1-st order autoregressive model of the fMRI time series (dynamic measures)
Multivariate modeling and predictive analysis	The multivariate association model of Ge et al., (2016) includes fixed effects (covariates; see above) and random effects (variability of phenotypic measures)
